# Sundry Facts, Opinions and Suggestions Derived from the Observation of Forty Cases of Tapeworm

**Published:** 1879-09

**Authors:** John Bartlett

**Affiliations:** Chicago


### ARTICLE IV.

### SUNDRY FACTS, OPINIONS AND SUGGESTIONS DERIVED FROM THE OBSERVATION OF FORTY CASES OF TAPEWORM. A Paper read before the Chicago Medical Society. By JOHN BARTLETT, M.D., of Chicago.

MR. PRESIDENT: — I have been led to believe that a summary of my experience, derived from the observation of a number of cases of tapeworm, would be acceptable to this Society. Accordingly I have jotted down, necessarily in a somewhat desultory manner, such facts, opinions and suggestions as seem of interest.

It is stated, in all accounts that have fallen under my notice, that the very great majority of tapeworms seen in this country are of the variety known as tænia solium. Just the contrary is my experience. Of about 55 tapeworms examined by me, 54 were T. mediocanellata, and one, T. lata. It is also stated that most frequently in this country the worm originates from the use of measly pork. In almost every case that I have treated, the patients stated that they were in the habit of eating rare beef, and that they seldom ate pork.

The length of the worm, it appears to me, has been grossly exaggerated. We hear of worms several hundred feet in length; and in the daily papers I have read of 600 feet of worm being extruded. To account for the disagreement in observers as to the length, I note first, that the tapeworm is very elastic, and that it has the power of contracting and extending itself.

Thus the proglottides, the segments which pass the body, elongate and contract, so that at one moment they are eight times longer than they are broad; at the next instant they are twice as broad as they are long; and the neck, which is generally as stated by writers, threadlike, sometimes shortens and thickens remarkably, as in the specimen before you. Now it is possible that a worm may be at one time 25 feet long, and at another 50; but I have never seen one which measured more than 25 feet when disentangled and laid out on the floor, and I think the majority will fall short of that measurement. As to the very long worms which have been reported, I incline to think that they may be likened to the zeuglodon of Col. Wood's Museum. This petrifaction was genuine, doubtless, but its vertebræ, which coiled indefinitely around the hall, represented pieces from a whole family of zeuglodons. To my mind, there is little doubt that the very long tapeworms of authors have been constructed in a somewhat similar manner.

All the heads of mediocanellatæ which I have seen, but one, were black. The younger the worm — and I date its age from the length of time the patient has had it — the smaller, to a certain age, is it; and the head of one only a few months old is smaller, less dark, less firm, and less definitely defined, than that of older ones. In one or two instances, when the worm was quite young, I have hesitated to say whether the object I was regarding was the head, or not, till the lens had been used. It is, however, usually easily recognized; it is as large as the head of a pin, and can be seen fifteen feet off, by the light of a tallow dip.

There would appear to be a considerable degree of misconception regarding the head of the tapeworm. Thus, regularly educated physicians have assured my patients that the head was as large as a kernel of corn; that it was about the size of a grain of wheat; that if the head I had obtained was visible to the naked eye, it was not a genuine find, for the true head was a microscopic object; and in another instance the doctors "had taken the worm away to look for the head with the microscope."

The supposition of Bemser, that the tæniæ solæ by nature drop their hooks, and thus, being incapable of retaining their hold in the intestine, are discharged, seems to me erroneous. Certainly, what we know of the tenacity with which the hookless worms retain their place, would indicate that the hooklets are unnecessary, so far as retention of position in the intestines is concerned.

*Manifestations of Vitality.* — In cases where the vitality of the parasite was not too much destroyed, it was quite active, and might seize, by its suckers, any surface near it. Often I have observed it fastened to the wider surface of its body; once it adhered to the side of the vessel in which I was attempting to drop it, and once it was found strongly holding to the side of the anal aperture as the patient arose from the vessel. On one occasion a rude ttempt to dislodge the head when so adhering, by drawing upon the neck at some distance from the head, resulted in a laceration of the neck, the head remaining fixed to the surface of attachment. In this instance it is interesting to note that the rupture took place at the juncture of the first joint of the neck with the head. Possibly, in the plans of nature to protect the parasite, this joint is made weak that the head may survive the removal of the entire body.

A person in whose hands I once left a tapeworm floating in milk, assured me that having kept the fluid at the temperature of the hand for some time, the worm finally imbibed it, swelling up round like a whip-cord. I should add that I do not place entire confidence in this statement; certainly I have seen nothing of the sort where the milk has been kept warm for half an hour or more.

*Cause.* — In only one instance within my knowledge has tapeworm followed the exhibition of raw meat to infants. The late Dr. Wagner directed such food for a patient, warning the mother of the possible danger from tænia. In a few months the progottides appeared.

One case has come to my knowledge in which the direct communicability of the disease would seem to have occurred. A patient of mine visited a country house, and while there passed a large piece of worm. The couple visited had never seen a tapeworm, and when it was known to them that their guest had just been relieved of one, they were desirous of viewing the specimen. The patient therefore washed the parasite, and laid it upon the porch, whence it was presently swept into the yard. Upon the next visit of my client to the old couple, each had a tapeworm.

*Symptoms.* — In some, every symptom is absent, save the annoyance of passing the pieces, and the health is perfect. In others, serious symptoms assigned to the disease by writers are noticed. In a cursory manner I will state such of these as now occur to me as having fallen under my notice. The appetite may be increased; it may be lessened; perhaps more commonly it is variable, the patient eating but little for a number of days, and then feasting voraciously. Emaciation is in some cases marked, in others it is entirely wanting; headache, mental dullness, loss of memory and energy are observed. Pain in the bowels, generally, I think, referred to the right or left lumbar region, is quite common. Also, a sense as of something falling about in the abdomen — as of the gravitation of a knuckle of intestine, loaded with the worm, from one part of the abdominal cavity to a lower postion. A feeling of something crawling high up in the rectum; a sensation as of the worm coming up into the mouth, exciting nausea. A sense of engorgement of the throat, accompanied, it was claimed, by an actual enlargement of the neck, and ending in an hystero-epileptic attack. Violent grinding of the teeth during sleep. A peculiar viscid state of the saliva, especially in the early morning; the appearance of an eruption like acne about the throat and chin. In two instances the mental irritation incident to a knowledge of the worm's presence threatened the sanity of the patient.

The irritation about the anus is sometimes distressing; one patient said to me: "I have worked all day, for 17 years, taking but little pleasure, except in my books after tea. For all this time I have, especially as soon as I sat down to read, been tormented to exasperation with this symptom. It has been to me an evil which no one who has not suffered from it can estimate."

The most remarkable case of probable dependence of a "reflex action" symptom upon tapeworm that I have encountered, is the following. It will be seen that the connection of cause and effect is not direct, but perhaps as nearly so as we often find: A young man recently married came to me to report a most remarkable condition of aspermatism. He seemed perfectly well, and had not at the time, nor had he previously had, any sexual diseases or weakness. Recollecting only one case of this condition reported by VanBuren and Keyes, I was at a loss to what cause to assign the symptom, and as to what to do to relieve it. Finally, at a venture, I asked, "What is your occupation?" Answer, "A butcher." "Have you got a tapeworm?" "No; I reckon not. What is that?" Having explained to him the nature of the parasite, and the fact of the passage of segments; he continued, "Oh, yes, I've got that; I've had it a long time." I advised him to have it removed. Some months later he told me that a druggist had taken away the most of his worm, and that the old difficulty had afterward disappeared.

I have seen a number of patients who have suffered ten fold more from the medicines they have taken than from the tapeworm. The injury seems to have been done to the stomach and bowels. One patient stated that he was a year or more recovering from a large dose of turpentine, which had been taken without cure. Others complained, for more than a year after the removal of the worm, of a set of gastric symptoms, which had followed the use of a large quantity of carbolic acid as a vermifuge. Occasionally, however, symptoms so referred to the medicine are really not dependent upon any damage it has inflicted, but upon some associate diseased condition. Thus, in a case occurring in the hands of a prominent practitioner of this city, the doctor got the credit of having injured his patient by excessive medication, till an autopsy, revealing chronic atrophy of the liver, relieved him of the charge.

Patients and their physicians are too apt, when the existence of a tape worm is discovered, to refer whatever symptoms may be present to it, and occasionally disappointment results, because of the continuance of these after the removal of the worm. Thus, one of my patients had disease of the kidney in a masked form. After the riddance of the guest, the symptoms of renal disease stood out more prominently and received notice. Medical men should be cautious in expressing the opinion that the newly discovered parasite is the *fons et origo* of all the ills of their patients, and chary of promises of complete relief from them.

In one of my cases, where the head was obtained and mounted on a slide by her attending physician, symptoms of a second worm appeared in 11 months. And it is worth recording, that on or about the same day upon which this patient recognized the appearance of segments, her nephew, living with her, discovered that he also was affected.

The disease occurs, as might be presumed, in families. Thus,

I have known two sisters and a brother to be afflicted with it at the same time.

*Frequency.* — Tapeworm, up to the last 10 or 15 years, was a rare affection — so that I have met a number of gentlemen who have practiced a quarter of a century and never treated a case. In the French army, of 250,000 men, only seven were found to be affected with it. Of late years, and according to a Southern writer, since the war, it has become greatly more common. I doubt not that there now exists one tapeworm for every 1,000 of the population of this section of the country.

*Duration.* — Among the patients of which I have had knowledge, in two the tapeworm had endured 17 years, in one 20 years, and in another 35 years.

Probably any number of worms may exist at once. I on one occasion knew two to pass; at another time five were extruded — they were all diminutive, apparently half-grown, and of the same size.

*Diagnosis.* — It has been well said that the only true diagnostic symptom of tapeworm is the passage of the joints; but the question arises: Do the segments always pass in an individual having tapeworm? For a long time I had no doubt that this question should be answered in the affirmative, but of late years I rather incline to the opinion that segments do not always pass from those afflicted with a full-grown worm. A patient of mine assured me most positively that it was only at long intervals that she found them in her discharges. The supposition that they may not appear at all times is supported by the statement of writers that they pass in greater abundance during some months than others — as in February and March, and in October and November — and also from the fact observed by Kuchenmeister, in a dead body in which a tapeworm existed, namely, while in the lower bowels no proglottides were found, on the walls of the intestines was discovered a granular matter which proved to be the ova of tænia. In fact, Kuchenmeister states that the segments may disintegrate in the bowels. I presume, therefore, that the absence of joints in the stools is not absolute proof that no worm is present.

In several instances I have known patients passing segments of tapeworm to be treated for years by distinguished practitioners for thread-worms. In these cases the prescriptions had been made from the report of the patient alone.

In two instances in my practice, great alarm has been occasioned in households, because, as the mother declared, in calling for me, "The intestines of the child were all falling out." In these cases some six or ten feet of worm, doubled several times on itself, had escaped from the bowel.

Occasionally cases occur of what might be styled pseudo-tænia, in which the subjective symptoms of the presence of the parasite are well-marked, and yet entozoa are not present. The most conspicuous case of pseudo-tapeworm I ever saw was that shown me in St. Luke's Hospital several years since, by Dr. Heydock. The patient was a young woman, sent from a neighboring State. She presented a catalogue of symptoms which might be classed as those of tænia, and these additional: She had a firm conviction that she had within a monster worm or snake; most carefully she described its location, its motions, its behavior when hungry and demanding food, and with great particularity detailed the formation and color of its head, and the manner in which it opened and shut its mouth on her tissues. The other symptom was, a violent pain as from the grip of a pincer upon the inner coats of the intestines about in the site of the gall bladder. This symptom to the bystander was presented as follows: Very often, perhaps every ten minutes, during the 24 hours, sleeping and waking, the patient, however engaged the previous moment, would clasp her hand with force against the painful spot, assume the attitude and facial expression of one agonized with pain, and scream out so as to be heard a block away. She would use no article of food unless it was literally covered with red pepper, because when so peppered the worm did not torment her, as she found it to do when food was taken without the condiment.

The second patient was a well educated and very intelligent young man. His health had been poor for years. He presented almost every symptom of worms laid down in the books, and in the most aggravated form. Judging from these, this patient, of all others, would have been supposed to labor under tænia.

I can detail three symptoms only: It was a labor and pain at times to keep his eyes open; his appetite was perfectly insatiable, and if it was not immediately appeased, distress in the epigastric region became intolerable, so that he went nowhere without a basket of food. There was a decided interference with the respiratory function, involving, apparently, the diaphram and abdominal muscles at a moment of inspiration. Let the patient be never so intently talking, every five minutes he would arise, stand with the body inclined forward, and make a rotary motion of the trunk as if endeavoring to relieve the abdomen from the sense of the clothes, at the same time, judging from the expression of the face, the entire will energy was absorbed in an effort to inspire; this act being accomplished, the sufferer would sit down and proceed with his narration, to again halt and go through with the acts described the next five minutes.

Neither of these individuals had a tapeworm. I have met several other cases where the patient was tormented with the idea of having a worm or snake in the stomach to an insane degree.

*Prognosis.* — It is stated by some writers that though the head may not be found after treatment a cure may yet result. This has, in several instances, occurred in my practice, but it is not generally the experience of others, so far as I can learn. The impression that in cases where all comes but the head the latter is apt to die, is probably false. I am certain that at least 100 instances have been reported to me in which "all came but the head," and the worm reappeared in due time. The fact seems to be that when the head is not found and the worm does not reappear the head has either passed unobserved or been directly killed by the dose given.

*Remarks on Treatment.* — The male fern in the form of ethereal extract sometimes does well. Dr. Groesbeck, who reports four successful cases with it, gives one drachm of the imported extract at 9 a. m. and one at 10.30 a. m., in a cup of milk, on an empty stomach; others have used it in this way with like success.

My experience with this remedy is not so favorable. It seemed in some cases to act quite promptly, and to expel the worm broken in many pieces, but in three such trials the worm reformed.

Some give with success large doses, four and even six ounces of pumpkin seed in emulsion. I have prescribed it several times, in these doses, with very little disturbance to the patient and with the effect of bringing the worm entire, *except the head.* For delicate persons, and for children, it is the least hurtful of remedies, and it is as efficient as some more irritant articles. As a tentative means for determining whether tapeworm exists it is the best remedy. It can be made rather pleasant to the taste and it is not irritant to the stomach. I give it in two doses, two hours apart, followed in six hours by castor oil and honey.

In conversation with physicians as well as patients I have found no remedy to be used with so much confidence as kousso. But whatever article is given, care should be taken by the physician to thoroughly advise his patient how to take the medicine, and how to antagonize sickness. Instructions should be most emphatically given to him to this effect. "It is idle for you to take this medicine without following my directions exactly. Remember, when you have an operation to use a clean chamber, and after the evacution note if the worm be passing, if so, do not attempt to pull it out, but lie down on the floor beside the vessel till another operation occurs, or if one does not occur, take a large injection once, twice, three or four times, using, if you have it, doses of the worm medicine with the water. If the worm does not pass, send for me, letting it hang from you till I come. If circumstances render it necessary to remove the worm, draw upon it slowly and steadily. If once it is started never stop the motion; this would give the head opportunity to renew its hold; keep pulling till the very fine neck appears and then repeat the injection, and while the bowel is full of water, and if possible at the instant when its contents are discharged, draw again upon the worm, when it quite often will be washed out entire. Don't forget while you are waiting with the worm protruding to place something upon it, as the cover of the chamber, otherwise it may go back. When the worm is evacuated, make certain that no one empties the chamber before I come. Do not, out of idle curiosity, lift the worm up with a stick, or stir it, you may in this way break off and lose the head."

The better plan in the treatment of any case is to choose some day on which one expects to be comparatively at leisure, and to call often to note how the patient gets on.

It is commonly taught that it is better to give a purgative the night before the vermifuge is to be taken. I have often followed this practice, but I have discontinued it, except in cases where constipation exists. The object in giving this preliminary purgative was to empty the bowels, so that the vermifuge might come more directly in contact with the worm; and also to render the intestines more sensitive to the cathartic action of the medicine. Experience has caused me to believe that the evacuation of the bowels does not render the worm more exposed to medication. On the contrary, the fæces in the normal condition being virtually insoluble in the medicines given, do not dilute them, and by occupying space in the bowels, they actually leave less opportunity for the worm to escape the poison intended for it. Again, if the bowels be irritated over much by the preliminary purgative, the vermifuge acts so promptly, in some cases, that it seems to pass from the intestines before the worm has been exposed to it sufficiently long to secure its destruction.

When the action of the remedy is most satisfactory, the parasite comes *en masse*, in such volume as to cause the patient to be aware of something unusually large passing. Invariably the worm has been so tied into knots that I have not had the patience to disentangle it. If the result be not perfectly satisfactory, it comes out lengthwise, in the vast majority of cases the tail presenting, though in one case the head came first. When the worm thus passes in the direction of its length, it may happen that as operation follows operation, more and more will be extruded, till finally the head follows the rest. More commonly, when more or less has passed, extrusion ceases; and not unfrequently, if the worm be but moderately affected, it will re-enter the bowel. I have known many feet of it thus to re-pass the sphincter.

It sometimes occurs that in the *dernier ressort* of delivering the worm by force, when it has been advancing in a promising way, it suddenly ceases to pay out, leaving the sensation of a decided and rude obstruction; in some of these cases I am satisfied that the mass of the worm in a knotted condition has been pulled down against the sphincter, and is there arrested. It might be brought out by an injection, but that failing, I would introduce the finger, with the hope of encountering the mass, and with a purpose of turning it out, as one removes a placenta from the womb after abortion.

Sometimes, when attempting to withdraw a worm, it seems that part of the resistance is due to the clamping of the anus on the body of the parasite; in such a case, the sphincter might be distended to advantage with a suitable instrument, as a pair of bullet forceps.

When the worm is passed, if there is reason to believe that any medicine remains in the stomach — if, for instance, we have within the past two hours given a dose, and if the patient be distressed by it, as by nausea, colic, oppression of the head, etc., we should cause vomiting by simple means — as by drafts of warm water, titillation of the fauces, etc. — with the view of shortening the distress of the patient.

The detection of the head, if it be yet upon the worm, is seldom difficult. Generally it may be recognized in one minute by holding the worm in the hand after washing in clean water, and this, by tracing down the fine neck till the head is reached. Occasionally, on account of its resting in a fold of larger portions of the worm, the head has escaped observation for some minutes. It is better, where difficulty is experienced, to place the parasite on a marble slab or large dish, and proceed regularly to go from one end to the other, looking all over both surfaces of every segment, lest the head may have been detached by violence, and still remain adherent to some portion of the body. In case the worm is broken into pieces, there is no way to find the head but by straining the entire contents of the chamber through a coarse cloth. This is a most unsatisfactory plan, as there exist in the discharges numbers of objects which to a provoking degree resemble, at a glance, either the neck or head.

*Dose for Children.* — I had assumed that, in prescribing tapeworm remedies for children, a pro rata dose should be given, but upon reflection, I became convinced that as it was the worm for which the medicine was designed, the pro rating of the dose was not desirable, and in case the quantity of medicine given was so reduced, no curative result could be hoped for. In other words, it seemed to me apparent that however small the host, the fact that we were "doctoring" the worm and not the patient, should not be lost sight of. Experience has led me greatly to modify this opinion; for I learned that success followed smaller doses of tæniafuge, than *a priori* calculation would have indicated. I at first accounted for this fact by supposing that the intestines of the child being smaller, the medicine was applied more certainly and more directly to the entozoon. But subsequent experience has led me to adopt the apparently irrational opinion that in practice the dose should be adjusted for the patient according to weakness, age, susceptibility to cathartic action, etc. For results seem to show that if the patient be affected by the smaller dose, the worm will be removed by it.

The nervous symptoms produced by some of the remedies we use, as headache, pain in the limbs, confusion of sight, etc., seem to me to be dependent solely on intestinal irritation; for let the patient vomit a residue of the medicine and pass more by the bowel, and he will immediately find great relief.

Most all the remedies used for tapeworm, induce more or less nausea and vomiting, and indeed, in some persons, the main obstacle to the cure is their inability to retain the medicine.

By accident I discovered an admirable means of overcoming this difficulty in many cases. A very delicate lady, having taken a vermifuge became most distressingly sick at the stomach, and in consequence marked prostration ensued; as in this case the presence of the parasite was seriously impairing the health, it was particularly desirable that the medicine be retained; accordingly, having used other means, I inquired for the bay rum or cologne with which to bathe the temples, when the lady earnestly inquired if she might not use chloroform. I immediately caused her to inhale a little, and after a few inspirations, she said, "It is going away, the nausea, I mean. I can feel it sink down lower and lower into the stomach, and when I get a little more under the influence of the chloroform, it entirely disappears." The patient held the anæsthetic in her hand, and as needed by the sickness, she inhaled more or less. I have several times since used chloroform for a similar purpose, and always with success, though experience suggests that unless one is watchful, the sickness may seize the patient so suddenly that time is not allowed to effect a sufficiently decided influence of the anæsthetic. And I have reason to think that it will not succeed in all cases. One lady fell under my care by whom many remedies had been swallowed; but none had been retained more than a few minutes. Before she had taken one-half of the dose I offered her, the regurgitant action of the stomach threatened. I sat by her for three hours using chloroform against nausea, something as we do against labor pains, though giving really very little, and finally, the worm passed away.

It is the general impression among medical men, that in the act of vomiting the pylorus is closed, and that the stomach is emptied of its contents through the œsophagus alone. My experience with tapeworm remedies has demonstrated positively, that in regard at least to these medicines, this is not so; but that at each act of emesis a portion of the ingesta is thrown into the intestines. The fact upon which I base this statement is, that in some cases, where every dose was quickly vomited, the worm yet passed; and upon careful examination of the quantity of the remedy thrown off, the conclusion was reached that some was retained, while the act of vomiting was so vigorous, the medicine being sometimes ejected across the room, and so repeated, that it could not be assumed that the stomach contained any. And this idea is consistent with other facts. Thus, a patient suffering long and severely from extreme gastric irritability during pregnancy, seems for many months to eject every thing eaten; and the same may be said of some children with whooping cough; and yet vitality incompatible with the idea that all food has been rejected, remains. I doubt not that in these cases a quantity of chyme escapes into the intestine. If the position here taken be true, it has a bearing upon the treatment of certain forms of poisoning, by emesis. If this very act is liable to inject some of the poison into the bowels, the stomach pump should be preferred.

There is reason to believe that it is easier to secure an entire worm than only a short piece. Therefore, after an unsuccessful attempt, it may be better to wait till the worm has re-formed, before a second effort at treatment is made. I have, however, twice known success to follow remedies given when the remaining portion was not more than five feet in length.

